# Using “Big Data” to Capture Overall Health Status: Properties and Predictive Value of a Claims-Based Health Risk Score

**DOI:** 10.1371/journal.pone.0126054

**Published:** 2015-05-07

**Authors:** Rita Hamad, Sepideh Modrek, Jessica Kubo, Benjamin A. Goldstein, Mark R. Cullen

**Affiliations:** 1 General Medical Disciplines, Stanford University, Palo Alto, California, United States of America; 2 Quantitative Sciences Unit, Stanford University, Palo Alto, California, United States of America; Örebro University, SWEDEN

## Abstract

**Background:**

Investigators across many fields often struggle with how best to capture an individual’s overall health status, with options including both subjective and objective measures. With the increasing availability of “big data,” researchers can now take advantage of novel metrics of health status. These predictive algorithms were initially developed to forecast and manage expenditures, yet they represent an underutilized tool that could contribute significantly to health research. In this paper, we describe the properties and possible applications of one such “health risk score,” the DxCG Intelligence tool.

**Methods:**

We link claims and administrative datasets on a cohort of U.S. workers during the period 1996–2011 (N = 14,161). We examine the risk score’s association with incident diagnoses of five disease conditions, and we link employee data with the National Death Index to characterize its relationship with mortality. We review prior studies documenting the risk score’s association with other health and non-health outcomes, including healthcare utilization, early retirement, and occupational injury.

**Results and Conclusions:**

We find that the risk score is associated with outcomes across a variety of health and non-health domains. These examples demonstrate the broad applicability of this tool in multiple fields of research and illustrate its utility as a measure of overall health status for epidemiologists and other health researchers.

## Background

Researchers across many fields often struggle with how best to capture an individual’s overall health status, with options including both subjective and objective measures. Simple self-report measures have proven to be surprisingly predictive of mortality, often more so than objective measures of health [1,2]. Yet for investigators who are unable to collect survey data due to the expense, or for those with access to only secondary data sources, such measures are not available for use.

With the increasing availability of “big data” sources in the form of linkable digitized claims and administrative records, epidemiologists and health researchers now have the opportunity to conduct studies using large longitudinal datasets, such as those from Medicare. Such claims data are increasingly used in academic research settings to determine outcomes such as health diagnoses and medication adherence [3,4]. A potential advantage of claims data is their ubiquitousness and relatively low costs, as they require little or no additional data collection. Yet the sheer volume of records and number of entries may pose a challenge for those seeking to condense an individual’s chart into one marker of overall health status. In this context, the predictive algorithms—or “risk scores”—developed in corporate settings are particularly valuable. These scores were initially used by actuaries and insurers to create predictive algorithms to forecast health expenditures [5,6] and by the Centers for Medicare and Medicaid Services (CMS) to determine payments to health maintenance organizations [7]. Yet they have tremendous potential to be useful in population health, health economics, and other fields of research. Studies suggest that these algorithms are better at predicting health expenditures compared with simple measures of number of comorbidities or functional status [8,9]. Earlier objective measures relied on simply abstracting the seriousness or number of medical conditions from an individual’s medical chart [2], while these risk scores employ more complex algorithms.

There are a handful of risk scores that have been adopted in a limited fashion by academic health researchers. The algorithm inputs differ in each, including the use of prescription drug claims data [10], inpatient and outpatient diagnostic codes [11,12], or some combination of these in addition to healthcare utilization data [13,14]. These risk scores have been used primarily in health services research, particularly in studies of the U.S. health insurance market [15–18] and to predict health expenditures [19,20].

Given the increasing availability of claims data and the limited predictive value of prior objective measures of health, risk scores represent an underutilized tool that could advance the sophistication of health research. In this paper, we describe the properties, predictive value, and possible applications of one such risk score to formally introduce this novel metric to the academic health research community. Our goal is to demonstrate that such risk scores are valuable objective markers of overall health status for health researchers with access to claims data. We show that this risk score is predictive of a range of diverse short-term and long-term health outcomes, including mortality, as well as several non-health outcomes, demonstrating its broad applicability in health research. By illustrating its associations with a wide variety of health outcomes, we demonstrate its utility as an objective marker of overall health status that can be used in future studies that employ claims data.

## Methods

### Risk Score

We employ the DxCG Intelligence software produced by Verisk Health, which implements the Diagnostic Cost Group Hierarchical Condition Category (DxCG-HCC) models [21]. Verisk markets this classification system to employers, health plans, and others, as a medical management tool for the development of clinical intervention and quality programs and as a method to forecast expenditures and utilization. This score is computed using an individual’s age and gender, as well as Current Procedural Terminology (CPT) and International Classification of Diseases (ICD) codes and use of healthcare services from the previous year. These inputs are then used to predict an individual’s health expenditures in the coming year. The scores are standardized such that a score of 1 indicates that the individual’s health expenditures are likely to fall at the mean in the following year, in a nationally representative population defined by Verisk. Each unit increase predicts a one-fold increase in expenditures above the mean. The specific inputs into the predictive model developed by Verisk are proprietary and not described in this manuscript.

### Study Sample

The sample in which we demonstrate the properties and predictive value of this risk score is a cohort of manufacturing workers at Alcoa, a large U.S. employer for whom we have complete claims data. This includes all individuals who were working at the firm on January 1, 1996 with at least one risk score calculated during the period 1996–2011 (N = 14,161). This longitudinal dataset contains repeated observations per person, ranging from 1 to 16 depending on when an individual drops out of the sample. This yields 151,931 risk score (person-year) observations during this time period, or an average of 10.7 years per person. Observations with missing values in a given year are omitted from the relevant analyses. By 2011, there are 5,962 individuals remaining in the sample. We link these data with other datasets, including personnel and administrative information provided by Alcoa (Table 1). While this sample is not representative of the U.S. population or the U.S. workforce, we selected these individuals because of the extensive data available for this population that enable us to conduct the analyses we present here, and because these employees are all covered by similar insurance plans with comprehensive benefits, so that findings will not be confounded by insurance status.

**Table 1:** Linked datasets employed in this study.

Dataset	Contents
Personnel	Age
	Race
	Gender
	Employment status (e.g., active, on leave)*
Claims	International Classification of Diseases codes
	Current Procedural Terminology codes
	Dates of healthcare encounters
National Death Index	Date of death
Eligibility	Insurance status

* This variable was used to determine which employees to include in our sample, i.e., those who were actively employed on January 1, 1996.

### Health Conditions

We use the claims data to identify incident (i.e., new) cases of five disease conditions: diabetes, hypertension, asthma/chronic obstructive pulmonary disease (COPD), depression, and ischemic heart disease (IHD). For each of these conditions, individuals with one or more inpatient claims or two or more outpatient claims with a relevant ICD diagnosis code in a 365-day period are considered to have a new diagnosis of the disease in question. To rule out prevalent (i.e., pre-existing) cases, we require the individual to have no claims related to the diagnosis for the first two years of the study period. As our dataset includes claims data beginning in January 1, 1996, for each disease we exclude individuals with diagnoses in 1996–1997, such that the earliest possible date of diagnosis for a given disease is January 1, 1998. If the disease diagnosis is established based on two outpatient claims, the date of diagnosis is considered to be the date of the first claim. On the other hand, if an individual has two outpatient claims separated by more than 365 days early during the study period, and subsequently has two claims in the same year later during the study period, the date of diagnosis will be based on the later claims, as the first two claims do not meet the criteria for diagnosis. This strategy, while imperfect, is similar to methods frequently used with claims data, and is unlikely to affect our study findings [4,22].

To examine mortality, we link our dataset with the National Death Index to obtain the date of death for individuals in the sample who died (N = 1,155), including those who left Alcoa at any point during the study period.

### Data Analysis

We conduct several analyses to illustrate the properties of the risk score, to examine its demographic correlates, and to demonstrate its relationship to a variety of health and non-health outcomes.

First, we present the risk score’s overall distribution and examine individual-level inter-class correlation coefficients and year-to-year correlation. To examine the extent to which the risk score is correlated with age, race, and gender, we conduct multivariable linear regression with individual-level random effects, clustering robust standard errors at the individual level to account for interdependence of the observations. We also control for year to adjust for secular trends.

We employ linear probability models with individual-level random effects to identify the degree to which an individual’s risk score in a given year is associated with the probability of being newly diagnosed with a disease condition in the following year. For example, an individual’s risk score in 2003 is used as the predictor variable for health outcomes in 2004; risk scores in 2011 are therefore not included in these analyses, as our dataset does not include health outcomes beyond 2011. As described above, these conditions include diabetes, hypertension, asthma/COPD, depression, and IHD. These analyses control for age, gender, race, and a dummy variable for each year to account for secular trends. Standard errors are clustered at the individual level to account for interdependence of the observations.

To assess the risk score’s association with long-term disease outcomes, we perform a time-to-event analysis for each of the disease conditions. Using the personnel dataset, we identify the last date that each individual was active at the firm, after which that individual is censored. We present unadjusted Kaplan-Meier survival curves by risk score quintile. We then estimate Cox proportional hazards models, controlling for race, gender, and age group (20–30 years old, 30–40 years old, etc.). We estimate two sets of Cox proportional hazards models: (1) with risk score as a continuous variable, and (2) with risk score by quintile.

For mortality analyses, we present unadjusted Kaplan-Meier survival curves by risk score quintile in 1996. The National Death Index includes deaths through September 1, 2011, after which we censor surviving individuals. We estimate Cox proportional hazards models, controlling for race, gender, and age group. As above, we estimate two sets of Cox proportional hazards models: (1) with risk score as a continuous variable, and (2) with risk score by quintile.

### Ethics Approval

The Stanford University Institutional Review Board provided ethics approval for this study (Protocol 16281). Individual informed written consent was waived by the Institutional Review Board based on an epidemiological exemption.

## Results

### Risk Score Properties

**Fig 1:**
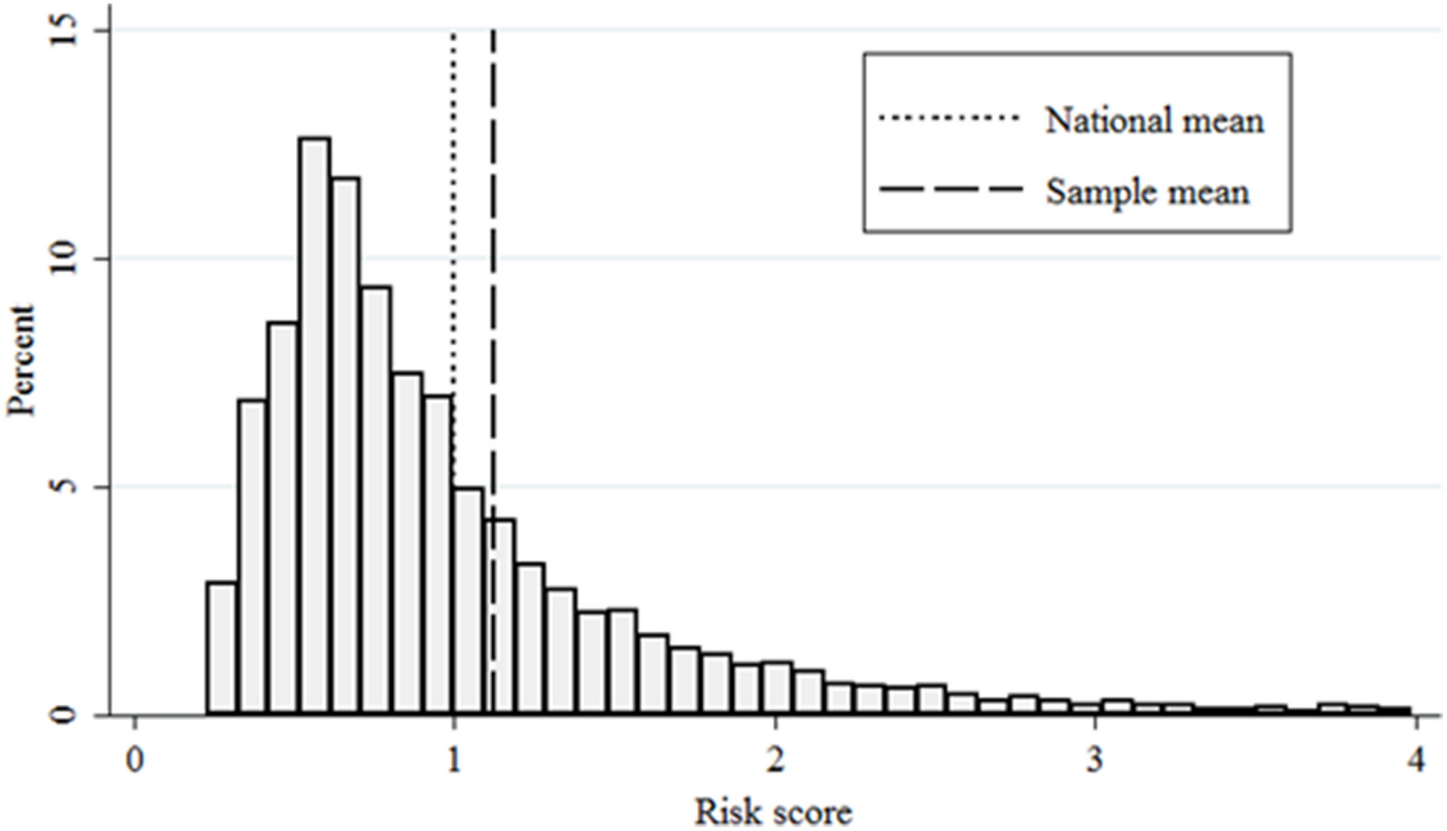
Risk score distribution in 1996. Note: For clarity of presentation, we omit observations with a risk score of greater than four (2.1%). Sample includes individuals employed at the firm on January 1, 1996 with at least one risk score during 1996–2011(N = 14,161).

The mean risk score in this population in 1996 is 1.12, with a standard deviation of 1.36 (Table 2). Fig 1 illustrates the distribution of the risk score in this population in 1996. For clarity of presentation of this figure, we omit those with risk scores greater than four, representing 2.1% of individuals (with mean and maximum scores of 7.73 and 33.19, respectively).

**Table 2:** Sample characteristics.

**Sociodemographic characteristics.**	
Female (%)	10.1
Age in 1996 (mean ± SD)	46.7 ± 8.7
Race (%)	
White	87.1
Black	8.5
Hispanic	3.7
Other	0.7
**Health characteristics**	
Deaths during 1996–2011 (%)	8.2
New disease diagnoses during 1996–2011 (%)	15.9
Diabetes	39.6
Hypertension	6.6
Asthma/COPD	4.7
Depression	14.6
**Risk score properties (1996)**	
Mean ± SD	1.12 ± 1.36
Median	0.79
Min, Max	0.23, 33.19
Quintiles	
Q1	0.23, 0.53
Q2	0.53, 0.69
Q3	0.69, 0.93
Q4	0.93, 1.38
Q5	1.38, 33.19

Inclusion criteria: Employed on January 1, 1996 with at least one risk score in the period 1996–2011 (N = 14,161). COPD = chronic obstructive pulmonary disease.

Risk scores are fairly stable over time. Year-to-year correlation for an individual is 0.49, with an individual-level inter-class correlation coefficient of 0.67. That is, 67% of the observed variance is between rather than within individuals. We next examine demographic factors that are correlated with the risk score (Table 3). Risk factors for higher risk score include age (β = 0.51 per 10-year increment, p < 0.001), being female (β = 0.12, p = 0.005), and being black (β = 0.45, p < 0.001). After controlling for these covariates, we observe an annual increase in the average risk score of 0.025 units during the study period (p < 0.001). Given the aging of the sample and these secular trends, the mean risk score in 2011 is 1.83 with a standard deviation of 2.72.

**Table 3:** Risk score correlates.

Variable	Beta	[95% CI]
Age (per 10-year increment)	0.51**	[0.48, 0.54]
Female	0.12**	[0.03, 0.20]
Race (Ref = White)		
Black	0.45**	[0.31, 0.59]
Hispanic	0.078	[-0.085, 0.24]
Other	-0.14	[-0.51, 0.22]
Year	0.025**	[0.021, 0.030]
Constant	-51.74**	[-61.06, -42.41]
Observations	151,931	
Individuals	13,880	

* p <0.05,

** p < 0.01.

Note: Sample includes individuals employed at the firm on January 1, 1996. Analysis conducted using multivariable linear regression with individual-level random effects. Robust standard errors are clustered at the individual level.

### Associations with Disease and Mortality

Each increment of 1 in the risk score is associated on average with an increased likelihood of receiving a new diagnosis of asthma (0.04%, p < 0.001), depression (0.02%, p < 0.001), diabetes (0.05%, p < 0.001), and IHD (0.04%, p < 0.001) in the following year (Table 4).

**Table 4:** Associations between risk score and new disease diagnosis in subsequent year.

	Coefficient [95% CI]				
	Asthma	Depression	Diabetes	Hypertension	Ischemic heart disease
Previous year risk score	0.00041**	0.00021**	0.00047**	-0.000077	0.00041**
	[0.00026, 0.00057]	[0.000097, 0.00033]	[0.00026, 0.00068]	[-0.00037, 0.00022]	[0.00019, 0.00063]
Age	0.000024	-0.00017**	0.00018**	0.00027**	0.00050**
	[-0.000015, 0.000063]	[-0.00020, -0.00013]	[0.00013, 0.00023]	[0.00018, 0.00035]	[0.00045, 0.00055]
Female	0.0023**	0.0025**	-0.0030**	-0.0030**	-0.0047**
	[0.00098, 0.0036]	[0.0013, 0.0037]	[-0.0044, -0.0017]	[-0.0052, -0.00076]	[-0.0058, -0.0037]
Race (ref white)					
Black	-0.00072	-0.00093*	0.0067**	0.010**	-0.0010
	[-0.0019, 0.00048]	[-0.0018, -0.000030]	[0.0045, 0.0089]	[0.0073, 0.013]	[-0.0027, 0.00064]
Hispanic	-0.0026**	0.000030	0.0052**	0.0023	-0.00071
	[-0.0038, -0.0014]	[-0.0015, 0.0015]	[0.0022, 0.0082]	[-0.0014, 0.0060]	[-0.0030, 0.0016]
Other	-0.0021	-0.0010	0.00090	-0.0062	-0.0017
	[-0.0055, 0.0012]	[-0.0043, 0.0023]	[-0.0056, 0.0074]	[-0.014, 0.0020]	[-0.0069, 0.0034]
Observations	143,822	144,392	139,633	127,321	141,706
Individuals	13,681	13,736	13,293	12,191	13,441

* p < 0.05,

** p < 0.01.

Note: Sample includes individuals employed at the firm on January 1, 1996. Analyses are conducted using linear probability models with individual-level random effects, in which an individual’s risk score in one year predicts their likelihood of a new diagnosis of disease in the following year. Standard errors are clustered at the individual level. To be considered a new diagnosis, the individual must have been free of the disease for the first two years of the study. For each of these conditions, individuals with one or more inpatient claims or two or more outpatient claims with a relevant ICD diagnosis code in a 365-day period are considered to have the disease in question. Each model includes dummy variables for year to control for secular trends.

**Fig 2:**
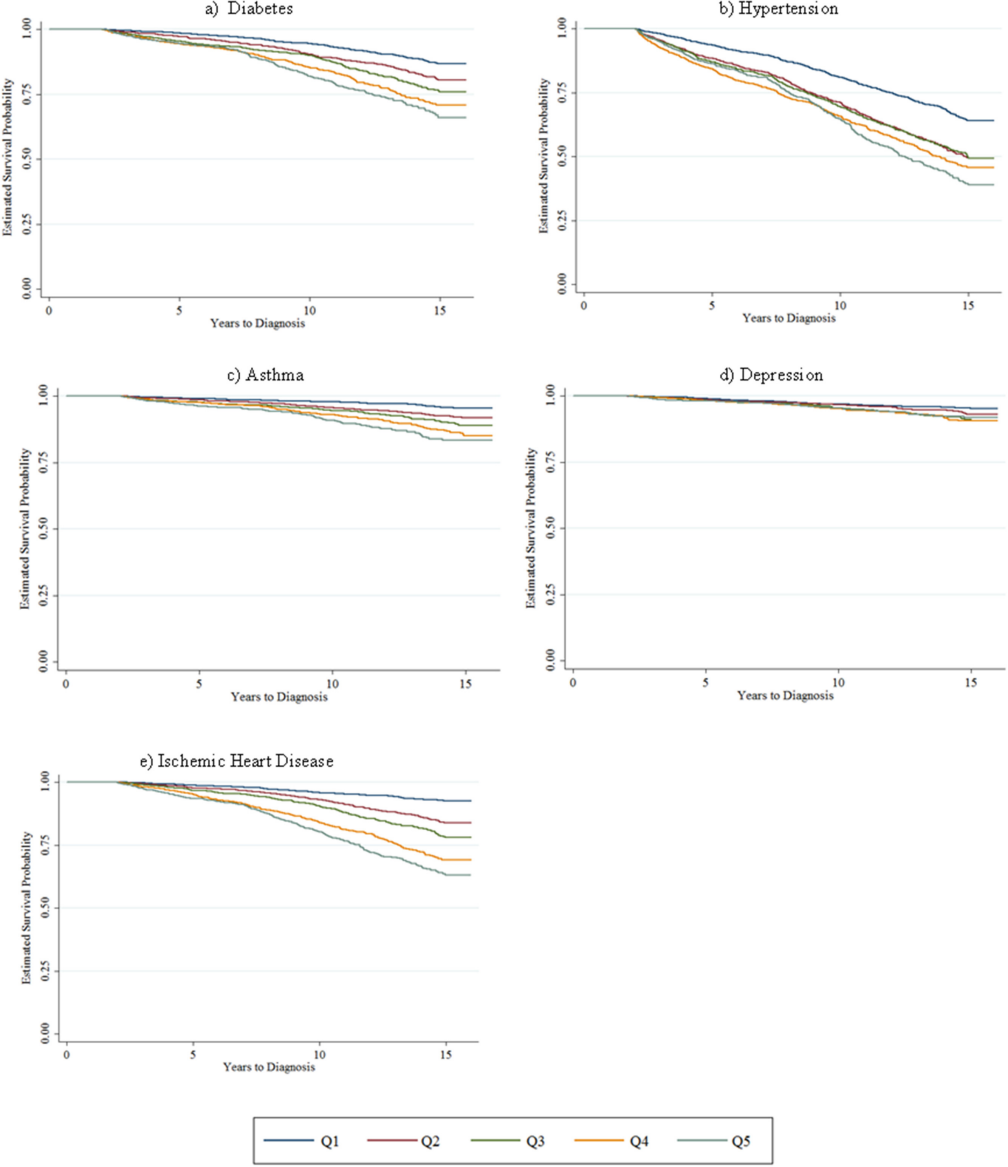
Kaplan-Meier survival curves for chronic disease diagnoses, by 1996 risk score quintile. Note: Sample includes individuals employed at the firm on January 1, 1996. For each of these conditions, individuals with one or more inpatient claims or two or more outpatient claims with a relevant ICD diagnosis code in a 365-day period are considered to have a new diagnosis of the disease in question. To rule out prevalent (i.e., existing) cases, we require the individual to have no claims related to the diagnosis for the first two years of the study period. As our dataset includes claims data beginning in January 1, 1996, for each disease we exclude individuals with diagnoses in 1996–1997, such that the earliest possible date of diagnosis for a given disease is January 1, 1998. N = (a) 8,522; (b) 7,641; (c) 8,841; (d) 8,886; (e) 8,665.

We next examine the risk score’s long-term predictive abilities, using the baseline value of the risk score in 1996 to examine time-to-diagnosis for each condition. Kaplan-Meier survival curves demonstrate a monotonic increase in likelihood of diagnosis for every condition with higher risk score quintiles (Fig 2). Cox proportional hazards models show that higher risk scores are associated with increased risk of asthma (HR 1.09, p = 0.001), diabetes (HR 1.09, p < 0.001), hypertension (HR 1.05, p = 0.007), and IHD (HR 1.10, p < 0.001) (Table 5). Using risk score quintiles as a predictor in Cox models confirms the relationship observed in the Kaplan-Meier curves, i.e., there is a monotonic increase in likelihood of diagnosis with higher risk score quintiles for most health conditions (Table 6).

**Table 5:** Cox proportional hazards models for incident disease diagnosis and mortality.

	Hazard Ratio [95% CI]					
	Asthma	Depression	Diabetes	Hypertension	Ischemic heart disease	Mortality
1996 risk score	1.09**	1.07	1.09**	1.05**	1.10**	1.21**
	[1.03, 1.14]	[0.99, 1.15]	[1.06, 1.13]	[1.01, 1.08]	[1.06, 1.14]	[1.19, 1.24]
Female	1.51**	1.63**	0.62**	0.83**	0.36**	0.67**
	[1.20, 1.92]	[1.26, 2.11]	[0.50, 0.77]	[0.73, 0.94]	[0.27, 0.50]	[0.50, 0.91]
Race (ref white)						
Black	0.76	0.85	1.81**	1.87**	0.94	1.29*
	[0.54, 1.07]	[0.56, 1.27]	[1.51, 2.18]	[1.64, 2.13]	[0.75 1.17]	[1.02, 1.63]
Hispanic	0.40**	1.09	1.91**	1.00	0.90	1.10
	[0.20, 0.78]	[0.65, 1.82]	[1.47, 2.49]	[0.81, 1.23]	[0.65, 1.25]	[0.74, 1.64]
Other	0.63	0.84	1.05	0.86	1.22	0.72
	[0.16, 2.52]	[0.21, 3.38]	[0.50, 2.23]	[0.47, 1.55]	[0.55, 2.72]	[0.27, 1.93]
Observations	8,841	8,886	8,522	7,641	8,665	9,012

* p < 0.05,

** p < 0.01.

Note: Sample includes individuals employed at the firm on January 1, 1996. To be considered a new diagnosis, the individual must have been free of the disease for the first two years of the study. For each of these conditions, individuals with one or more inpatient claims or two or more outpatient claims with a relevant ICD diagnosis code in a 365-day period are considered to have the disease in question. Each model includes dummy variables to control for age group at baseline (20–30 years old, 30–40 years old, etc.). Individuals were censored at the last date that they were active at the firm based on the personnel dataset. For mortality, individuals were censored at September 1, 2011, after which we do not have data on mortality.

**Table 6:** Cox proportional hazards models for incident disease diagnosis and mortality, by 1996 risk score quintiles.

	Hazard Ratio [95% CI]					
	Asthma	Depression	Diabetes	Hypertension	Ischemic heart disease	Mortality
Risk score quintile (ref = Q1)						
Q2	1.50*	1.39	1.18	1.31**	1.43**	1.32
	[1.09, 2.07]	[0.98, 1.95]	[0.97, 1.45]	[1.15, 1.48]	[1.12, 1.84]	[0.92, 1.91]
Q3	1.91**	1.96**	1.37**	1.20**	1.67**	1.08
	[1.39, 2.62]	[1.40, 2.74]	[1.12, 1.68]	[1.05, 1.36]	[1.31, 2.14]	[0.74, 1.56]
Q4	2.32**	2.28**	1.70**	1.39**	2.35**	1.35
	[1.69, 3.20]	[1.61, 3.22]	[1.38, 2.08]	[1.22, 1.59]	[1.85, 2.99]	[0.94, 1.94]
Q5	2.75**	2.18**	1.91**	1.43**	2.73**	2.24**
	[1.98, 3.81]	[1.50, 3.17]	[1.54, 2.37]	[1.24, 1.65]	[2.13, 3.49]	[1.57, 3.19]
Female	1.27	1.33*	0.56**	0.78**	0.31**	0.63**
	[0.99, 1.62]	[1.02, 1.74]	[0.45, 0.69]	[0.68, 0.88]	[0.23, 0.43]	[0.46, 0.85]
Race (ref white)						
Black	0.75	0.84	1.81**	1.86**	0.95	1.29*
	[0.54, 1.06]	[0.56, 1.26]	[1.51, 2.18]	[1.63, 2.12]	[0.76, 1.19]	[1.02, 1.63]
Hispanic	0.38**	1.07	1.92**	1.00	0.87	1.05
	[0.20, 0.76]	[0.64, 1.80]	[1.48, 2.49]	[0.81, 1.23]	[0.63, 1.21]	[0.70, 1.57]
Other	0.67	0.85	1.06	0.88	1.44	1.03
	[0.17, 2.70]	[0.21, 3.43]	[0.50, 2.25]	[0.48, 1.58]	[0.64, 3.21]	[0.39, 2.77]
Observations	8,841	8,886	8,522	7,641	8,665	9,012

* p < 0.05,

** p < 0.01.

Note: Sample includes individuals employed at the firm on January 1, 1996. To be considered a new diagnosis, the individual must have been free of the disease for the first two years of the study. For each of these conditions, individuals with one or more inpatient claims or two or more outpatient claims with a relevant ICD diagnosis code in a 365-day period are considered to have the disease in question. Models include dummy variables to control for age group at baseline (20–30 years old, 30–40 years old, etc.). Individuals were censored at the last date that they were active at the firm based on the personnel dataset. For mortality, individuals were censored at September 1, 2011, after which we do not have data on mortality.

**Fig 3:**
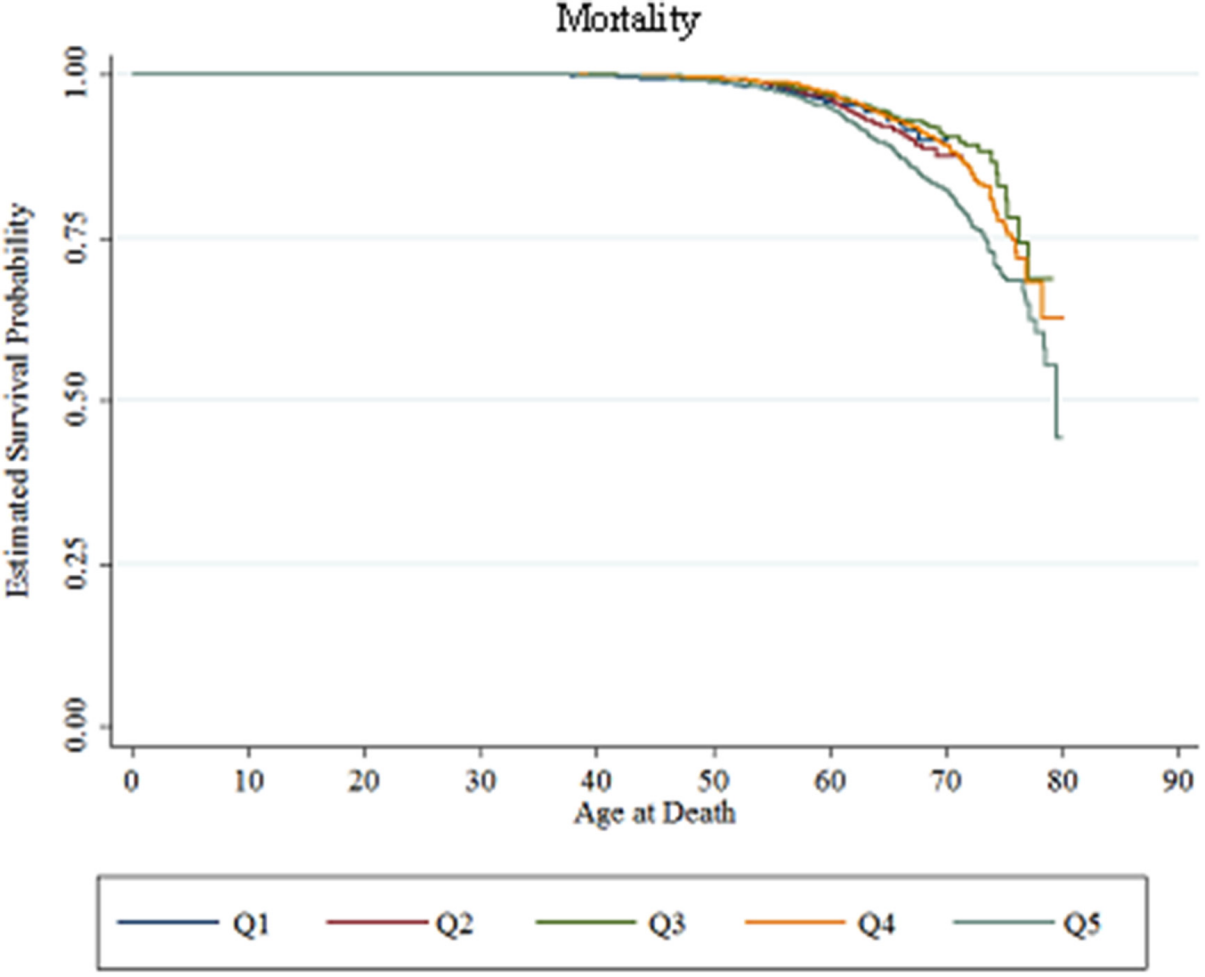
Kaplan-Meier survival curve for mortality, by 1996 risk score quintile. Note: Sample includes individuals employed at the firm on January 1, 1996. Individuals were censored at September 1, 2011, after which we do not have data on mortality. N = 9,012.

Similarly, we find that higher risk scores in 1996 are more strongly associated with mortality during the follow-up period (Fig 3), with a hazard ratio of 1.21 (p < 0.001) (Table 5). When using risk score quintile rather than continuous risk score as the primary predictor, we find that the relationship between risk score and mortality is largely driven by those in the highest quintile (HR 2.24, p < 0.001), the only group with a significantly elevated HR (Table 6). Within this top quintile, we find that individuals in the 95^th^-100^th^ percentile had a higher risk of mortality compared to those in the 80^th^-95^th^ percentiles (HR 2.38, p < 0.001) (data not shown).

## Discussion

The actuarial and insurance industries have long employed predictive algorithms to produce health risk scores for the purposes of medical management and cost prediction. In this paper we use one such risk score as a marker of overall health status for a broad array of applications in epidemiology. We demonstrate that the score displays within-individual stability across time. Even after controlling for age, race, and gender, the risk scores increase over time, which may reflect changes in physician coding behavior or secular health utilization trends. This risk score is associated with multiple short-term health outcomes. It is possible that this correlation reflects the increased utilization of healthcare that may immediately precede a new diagnosis, but we also demonstrate its predictive ability for several long-term health outcomes, including mortality at higher quintiles.

The size of the associations is modest, with likely limited clinical relevance at the individual level. The value instead lies in the risk score’s potential use as a marker of overall health status in research studies and in its short- and long-term prediction ability at the population level. Interestingly, we find that individuals in the second and third risk score quintiles at baseline demonstrate increased long-term likelihood of being diagnosed with several different diseases compared to the lowest quintile, even though they are healthier than average (with risk scores between 0.53 and 0.93). This provides evidence of the sensitivity of the risk score in identifying individuals at high risk of developing chronic disease, even at low values.

In prior research by our group, we have found that this risk score is associated with several other health-related outcomes. For example, in a study of the impacts of the Great Recession of 2007–2009 on healthcare utilization among a panel of employees, individuals with higher risk scores at baseline in 2006 utilized more outpatient, emergency room, and inpatient services at baseline, as would be expected based on the manner in which the risk score is constructed. However, after a period of several years in which there was reversion to the mean, individuals with higher risk scores responded to the recession with greater increases in utilization, compared with those with lower risk scores at baseline [23].

In a prior study examining predictors of complications among diabetic patients, higher risk score quartiles predicted increased risk of complications including coronary artery disease, stroke, heart failure, and renal disease [24].

Higher risk scores are associated with a variety of non-health related outcomes, illustrating the broad applicability of the risk score to multiple fields of research. For example, those with higher risk scores were more likely to be laid off during the Great Recession [22,25]. In another study, individuals with higher risk score deciles were more likely to experience occupational injury, even controlling for other demographic and job-related factors [26]. Those with higher baseline risk scores were also more likely to enter retirement at younger ages [27], and were more likely to have a delayed return to work after a hospitalization (unpublished).

A number of studies by other groups have found that other claims-based risk scores predict mortality, long-term care utilization, and 30-day readmission after hospitalization [28–30]. Risk scores have also been employed in studies of moral hazard, generalized risk aversion, and adverse selection in the U.S. health insurance market [17,18,31]. For implementation of causal g-methods, such as marginal structural models or g-estimation, the risk score could serve as a longitudinal measure of health status. In this case, it serves as a time-varying measure of health status to address the healthy worker survivor effect. This bias arises if workers in better health tend to accrue more exposure than less healthy workers who are more likely to transfer to lower exposed jobs or leave work. In most occupational studies, time off work has been the only available surrogate for health status to address this bias and is only weakly related to exposure and health. In a recent study of exposure to particulate pollution and IHD incidence in this workforce, the authors reduced this bias by applying marginal structural models and treating risk score as a time-varying measure of comprehensive health status [32].

While these examples are not intended to illustrate a causal role for the risk score, they demonstrate the broad utility of this measure across a variety of health and non-health domains, and in particular its utility in mitigating confounding by health status.

Our group has found that this software is simple to use. Verisk offers the package at a discount to academic researchers, making it accessible to those who wish to apply this versatile tool. As health researchers strive to take advantage of the increasingly available vast quantity of claims data, including those from Medicare and private insurers, employing risk scores presents the possibility of collapsing large quantities of data into one index of overall health status. This technique is also relatively inexpensive compared to surveys required to capture subjective measures, if claims data are readily available [19].

While researchers wishing to predict a particular health outcome as accurately as possible may consider developing their own predictive algorithm, this requires large amounts of data and expertise that may not be available. In contrast, for those interested in a broadly applicable measure of overall health status that is available “off the shelf,” existing risk score algorithms such as this one may be well suited for this purpose. Similarly, while a single composite measure is likely to explain less variance than multiple measures, in this case it is impractical to include the richness of an individual’s entire claims history as individual variables. Moreover, a composite measure accounts for fewer degrees of freedom.

Given the variety of claims-based risk scores produced in private settings and those available for public use (e.g., through CMS and the Agency for Health Research and Quality), researchers have several options in selecting amongst these tools. While a handful of prior studies have compared the predictive value of these risk scores for expenditures [6,7,19,33], fewer have compared their predictive values for health outcomes [34,35]. Moreover, it is likely that different measures are likely to predict different outcomes to varying degrees [36,37]. These are questions that can be explored in future studies.

This study has several limitations. The risk score we employ here is in part capturing health behaviors—i.e., willingness and ability to access healthcare services—rather than health itself. As we show, it is nevertheless associated with a variety of health outcomes. Given differences in the collection of claims data in other countries, the potential use of this particular risk score in international settings is limited. Additionally, such algorithms are often proprietary, meaning that the specific inputs that form the components of the risk score are unknown to those researchers who choose to use it. Finally, this study is limited in its application of this risk score among a non-representative sample of manufacturing employees. Employed individuals have been shown to be healthier than the general population [38], which limits the external validity of the specific findings that we describe here, and this sample has fewer women and minorities than the general population. The availability of extensive claims and other linkable data for this population, however, enabled us to conduct the diverse set of analyses we present here. Future studies should test this tool in other populations, including the non-employed, the elderly, and others.

In this paper, we describe the properties and possible applications of a claims-based health risk score, demonstrating its associations with mortality, incident disease diagnosis, and healthcare utilization, in addition to a range of non-health outcomes. These examples demonstrate the broad applicability of this tool across a variety of domains, and illustrate its utility as a measure of overall health status for epidemiologists and other academic health researchers.
